# Meetings of Societies

**Published:** 1924-01

**Authors:** 


### MEETINGS OF SOCIETIES.

### Bristol Medico-Chirurgical Society.

*November 14th*, 1923.

Mr. T. Carwardine gave an address on "**The Value of Skiagraphy in the diagnosis of duodenal ulcer.**"

In the discussion which followed the skiagraphist's technique was explained. Both screen and film examinations should be made in upright as well as recumbent positions. "Flash" exposures are necessary, to eliminate difficulties from visceral movement. A suggestion was made that a series of such flash exposures will in future be made, so that a kinematograph of the bowel is in effect taken.

Mr. Walters showed a case of **"Ligneous Thyroiditis."** He pointed out that the condition was very rare, was almost invariably diagnosed as malignant disease; that dissection of the growth was impracticable, as it very rapidly involved vessels and nerves of the neck; that a spontaneous recovery was the rule, and that X-rays were of assistance in this.

Mr. Hey Groves showed specimens from a case of **"Malignant growth of the bladder,"** treated by implantation of the ureters in the sigmoid colon and excision of the bladder, a three-stage operation. The patient was making a satisfactory recovery when she died of a pulmonary embolism.

Mr. Adams showed a case of **"Charcot's disease of both knee-joints in a woman."**

---

*January* 10*th*, 1924.

Dr. R. G. Gordon read a paper on **"The Difficult Child."** He defined the difficult child as one that from some cause or another cannot be made to fit into the normal environment, one who, suffering from no remediable physical disability, was not improved by ordinary school discipline —the child who could not adjust his ego to the normal environment. He proposed to consider especially children of school age.

The causes of the "failure of integration" which underlay this group of conditions were: (*a*) Inherent causes; (*b*) Disease; (*c*) Early training.

Integration depended on perfect cortical function, and its failure might manifest itself as: (1) Imbecility; (2) Mental deficiency; (3) Feeble-mindedness, Backwardness, Delinquency.

It was the last triple group that constituted the difficult child. For the others institutional provision was already made. The poor integration in this group led to the children being suggestible, easily led, and easily misled. There was further often a sense of inferiority, which led to shyness, crime, or apathy. Hero worship was very commonly a factor in increasing these troubles. Bullying, cheating, theft, and sexual disturbances were common manifestations of this condition in children at school.

The effects of disease must be considered. Epilepsy, congenital syphilis, encephalitis lethargica might each bring such manifestations in their train. Dr. Gordon cited cases of each.

With regard to inherent causes, Dr. Gordon especially deprecated the term moral defective. There was no such thing as a faculty of morality, and we ought not to speak as if there were. Very rare cases were those of complete psychopaths.

The lines along which the precocious child may develop were considered. Ability in school work or games were fortunate lines; devotion to fantasy, day dreaming, truancy and other delinquencies might be other manifestations of precocity.

The backward child was often a great difficulty. Many of these cases were merely, as the name implies, examples of delayed development, and did very well in later life.

Delinquency, *e.g.* stealing, truancy, was often exceedingly puzzling. Sexual disturbances must not be exaggerated in importance. There has been a tendency in this direction. Especially important was it not to use fear as a method of repressing masturbation. The psychic consequences of this habit might be unfortunate, but not nearly so damaging as the emphasis of a feeling of inferiority by attaching a special moral stigma.

Investigation of the actual condition of the child's mind was the first essential, putting aside all pre-conceived grown-up ethical and intellectual standards; a genuine and sympathetic attempt to understand the reason, however absurd a reason it might be, for the child's behaviour was the first step in helping him. Dr. Gordon agreed that many cases of difficulty were the consequence of early training. The individual child, however, was not helped by the opinion that he should have been differently brought up, and the problem he wished to consider was how to help the child that was already found to deviate from the normal. He considered that in many cases the solution would be found in special schools. Dr. Gordon cited cases to illustrate many of his more important points.

Dr. J. O. Symes, Dr. Alexander, Dr. E. C. Williams, and Mr. Whatley took part in the discussion.

